# Contribution of RND superfamily multidrug efflux pumps AdeABC, AdeFGH, and AdeIJK to antimicrobial resistance and virulence factors in multidrug-resistant *Acinetobacter baumannii* AYE

**DOI:** 10.1128/aac.01858-24

**Published:** 2025-05-23

**Authors:** Lulin Xie, Junwei Li, Qin Peng, Xianqing Liu, Fei Lin, Xiaozhen Dai, Baodong Ling

**Affiliations:** 1 Key Laboratory of Structure-Specific Small Molecule Drugs at Chengdu Medical College of Sichuan Province, School of Pharmacy, Chengdu Medical College 648168, Chengdu, China; 2 Department of Pharmacy, Clinical Medical College and The First Affiliated Hospital of Chengdu Medical College, Chengdu, China; 3 School of Biological Sciences and Technology, Chengdu Medical College, Chengdu, China; Universita degli studi di roma La Sapienza, Rome, Italy

**Keywords:** *Acinetobacter baumannii*, RND efflux pump, AdeABC, AdeFGH, AdeIJK, multidrug resistance, biofilm, virulence factor, pathogenicity

## Abstract

*Acinetobacter baumannii* is a critical priority gram-negative bacterial species characterized by multidrug resistance. The latter is significantly attributable to the resistance-nodulation-cell division (RND) superfamily of tripartite multidrug efflux systems represented by AdeABC, AdeFGH, and AdeIJK. By constructing isogenic deletion mutants, this investigation assessed the impact of RND efflux pumps on planktonic and biofilm cell antimicrobial susceptibility as well as on biofilm formation and virulence factors in a multidrug-resistant reference strain, *A. baumannii* AYE. Inactivation of individual genes encoding the aforementioned three RND pumps or regulators (i.e., AYE△*adeA*, △*adeB*, △*adeC*, △*adeRS*, △*adeFGH*, and △*adeIJK* mutants) demonstrated that the three efflux pumps, particularly AdeB, contribute to resistance in both planktonic and biofilm cells to structurally unrelated anti-*A*. *baumannii* drugs, including carbapenems, fluoroquinolones, macrolides, polymyxins, and/or tetracyclines/tigecycline. The pump inactivation also altered other functions, changes in bacterial motility and adhesion, reduction of biofilm formation, and decreased expression of the genes related to biofilm formation and virulence factors (*abaI*, *bap, bfmR*, *csuE*, *ompA,* and *pgaA*, except for *abaR* whose expression was increased). The virulence assay measured through the survival rates of *A. baumannii*-infected *Galleria mellonella* revealed the relation between RND pumps (particularly AdeB) and pathogenicity. The findings together expand the understanding of specific *A. baumannii* RND pumps or components for their roles in resistance and virulence/pathogenicity in the presence of high-level multidrug resistance, highlighting the RND pumps as potential therapeutic intervention targets against *A. baumannii* infection.

## INTRODUCTION

*Acinetobacter baumannii* is a major nosocomial pathogen around the globe and is also present in various environments (1–3). This microbe is featured with the production of various virulence factors and the presence of high-level intrinsic resistance to a range of antimicrobial drugs (3, 4). The latter provides an advantage for the microbe to survive in antimicrobial pressure environments such as hospitals (5, 6). *A. baumannii* can rapidly develop acquired resistance (7, 8). Indeed, the World Health Organization lists *A. baumannii* as one of the top critical priority pathogens that are of public health importance and require research, development, and strategies to prevent and control antimicrobial resistance (9). Epidemic isolates of *A. baumannii* (including fetal outbreak) with multidrug resistance or extensive drug resistance have been frequently reported worldwide (10–12). A U.S. national study with *A. baumannii* isolates from hospitals, long-term care, and outpatients reveals a multidrug resistance rate of 49% (13). In China, *A. baumannii* isolates also display a high prevalence (>50%) of resistance to a range of antimicrobials including carbapenems and reduced susceptibilities to biocides (14, 15).

The high levels of both intrinsic and acquired resistance of *A. baumannii* are attributable to a range of resistance mechanisms, predominantly including the alteration of drug targets, production of drug-inactivating enzymes, and the presence of drug efflux pumps (16, 17). Of these mechanisms, initially discovered in the 1990s in *Escherichia coli* and *Pseudomonas aeruginosa* (18–20), the active efflux process mediated by multidrug efflux pumps of the resistance-nodulation-cell division (RND) superfamily provides a unique ability for microbes against a variety of structurally-unrelated antimicrobial agents (4, 17, 21). Specifically, *A. baumannii* contains multiple RND efflux systems encoded by chromosomal genes (4) with three characterized systems, AdeABC, AdeFGH, and AdeIJK for their contribution to multidrug resistance (22–26). Each of these Ade RND pumps is typically a tripartite system containing an RND pump located in the cytoplasmic (inner) membrane (AdeB, AdeG, and AdeJ), a periplasmic adaptor/membrane fusion protein (AdeA, AdeF, and AdeI), and an outer membrane channel protein (AdeC, AdeH, and AdeK) (4, 17). These systems accommodate a large number of substrates covering β-lactams, aminoglycosides, macrolides, fluoroquinolones, phenicols, tetracyclines, and/or other toxic agents including biocides and disinfectants (4, 14, 27). These RND pumps are typically controlled by various local and global regulators, whose mutations can result in overexpression of RND pumps leading to elevated antimicrobial resistance (4, 17). For example, AdeABC is regulated by a two-component regulatory system encoded by AdeRS (28). Overproduction of RND systems yields enhanced multidrug resistance (25, 29–31).

RND pumps not only mediate antimicrobial resistance but also serve other functions such as bacterial stress response, fitness, colonization, and virulence (17). A major feature of *A. baumannii* relies on its pathogenesis, attributable to the production of various virulence factors and the formation of biofilm, which enable the microbe to adapt by adhering to and colonizing in any given environment (32, 33). Indeed, *A. baumannii* pathogenicity or virulence is evidently multifactorial (34). The accumulated evidence reveals the complexity of the functional roles of RND pumps in resistance and virulence (including quorum sensing and biofilm formation). For instance, AdeRS was found to regulate genes involved in multidrug efflux, biofilm formation, and virulence in a strain-specific manner (35). AdeFGH system influences the synthesis and transport of quorum-sensing signaling molecules, thereby promoting biofilm formation (36). However, data gaps exist in *A. baumannii* with respect to the roles of RND pumps in resistance and pathogenesis at different resistant levels.

To improve the understanding of Ade multidrug efflux systems, we aimed to investigate the effects of AdeABC, AdeFGH, or AdeIJK inactivation in a multidrug-resistant reference strain of *A. baumannii* on antimicrobial susceptibilities of planktonic and biofilm cells and the expressions of virulence factors. This approach allowed the determination of how the existing multidrug resistance and virulence gene expression could be affected by the absence of a major RND pump. The findings further support the clinically relevant importance of RND pumps in *A. baumannii* for both resistance and virulence.

## RESULTS

### RND pump expression and gene deletion confirmation

*A. baumannii* AYE is a strain used in literature for studying multidrug resistance (10, 35, 37–39). For comparison, we also included two frequently used reference strains, ATCC 17978 and ATCC 19606, which were isolated prior to the 1950s and are generally regarded as susceptible strains (40). First, RT-qPCR was conducted using primers (Table S1) to verify the expression of three tripartite RND pump systems, AdeABC, AdeFGH, and AdeIJK. The local two-component regulatory genes, *adeRS*, for regulating *adeABC* expression were also tested for their expression. The expression of 16S rRNA was used as the internal reference. As expected, the three RND efflux systems are constitutively expressed in the three tested strains (except for strain ATCC17978 lacking *adeC* in its genome) (Fig. S1). The expression of *adeR* and *adeS* was also readily detected (Fig. S1). These results are consistent with a previous study reporting the expression of *adeABC* and *adeIJK* of strain AYE (41). Our results also revealed the expression of *adeFGH*, which was twice lower than that of *adeABC*. Same with strain AYE, two ATCC strains also showed the highest expression with *adeJ* (for AdeIJK) (Fig. S1), whereas the expression of *adeABC* and *adeRS* in the AYE appeared between strains ATCC 19606 and 17978, and the expression of *adeG* in strain AYE was 4-fold or 2-fold higher than strains ATCC 19606 and 17978, respectively (Fig. S1). Together, the simultaneous expression of three RND pumps in strain AYE was observed, which supports the functional roles of the three systems in resistance and other functions. Therefore, the genetic deletion of the three RND pump system-encoding genes was conducted (using primers included in Table S2) to inactivate or change the relevant efflux systems. The isogenic deletion mutants generated in this study (i.e., strains AYE△*adeA*, △*adeB*, △*adeC*, △*adeRS*, △*adeFGH,* and △*adeIJK*) were confirmed by PCR (Fig. S2 and S3), followed by DNA sequencing.

### Antimicrobial susceptibility of planktonic cells

Antimicrobial susceptibility testing was conducted to measure the minimal inhibitory concentration (MIC) values of 15 antimicrobial drugs against planktonic cells of *A. baumannii* AYE and its six RND system gene deletion mutants. In particular, the individual gene inactivation of two operons, *adeRS-adeABC*, allowed us to assess the role of individual specific genes of the two-component system AdeRS-regulated tripartite AdeABC efflux system. The parental strain AYE showed, as expected, multidrug resistance to structurally different antimicrobial classes, including anti-*A*. *baumannii* agents such as third-generation cephalosporins (ceftazidime and ceftizoxime), fluoroquinolones (ciprofloxacin and levofloxacin), aminoglycoside (amikacin), chloramphenicol, and tetracycline (Table 1). According to clinical breakpoints available from the Clinical and Laboratory Standards Institute (CLSI) (42) and US Food and Drug Administration (43), the AYE strain is susceptible to carbapenems (imipenem and meropenem), polymyxin B, and tigecycline, but resistant to amikacin, ceftazidime, ceftizoxime, ciprofloxacin, levofloxacin, doxycycline, and tetracycline (Table 1). Although no clinical breakpoints were established for cefoperazone-sulbactam and azithromycin, the AYE strain is relatively susceptible to these antimicrobial drugs (with cefoperazone-sulbactam MIC of 16 µg/mL and azithromycin MIC of 8 µg/mL) but resistant to ampicillin and chloramphenicol (Table 1). The latter resistance observation is supported by the intrinsic resistance to ampicillin and chloramphenicol of *A. baumannii* that is established by CLSI (42).

**TABLE 1:** Effects of inactivating RND efflux system genes on antimicrobial susceptibility in planktonic cells of *A. baumannii*

Antimicrobial class	Antibacterial drug	MIC in μg/mL (Clinical breakpoint Susceptible [S], Intermediate [I] or Resistant [R]) for strain^*a*^						
		AYE	△*adeA*	△*adeB*	△*adeC*	△*adeRS*	△*adeFGH*	△*adeIJK*
β-Lactams	Meropenem	2 (S)	1 (S)	0.25 (S)	1 (S)	0.5 (S)	1 (S)	1 (S)
	Imipenem	2 (S)	1 (S)	0.5 (S)	1 (S)	0.5 (S)	0.5 (S)	1 (S)
	Ceftazidime	˃1024 (R)	˃ 1024 (R)	˃1024 (R)	˃1024 (R)	˃1024 (R)	˃1024 (R)	˃1024 (R)
	Ceftizoxime	˃1024 (R)	˃1024 (R)	1024 (R)	˃1024 (R)	˃1024 (R)	˃ 1024 (R)	1024 (R)
	Ampicillin	˃ 1024	˃ 1024	˃ 1024	˃ 1024	˃ 1024	˃ 1024	˃ 1024
	Cefoperazone-Sulbactam	16	8	8	16	8	16	16
Aminoglycosides	Amikacin	64 (R)	16 (S)	16 (S)	64 (R)	64 (R)	64 (R)	64 (R)
Fluoroquinolones	Ciprofloxacin	64 (R)	32 (R)	32 (R)	64 (R)	32 (R)	64 (R)	32 (R)
	Levofloxacin	8 (R)	2 (S)	2 (S)	8 (R)	4 (S)	8 (R)	2 (S)
Macrolides	Azithromycin	8	0.25	0.25	8	2	8	4
Phenicols	Chloramphenicol	1024	128	128	1024	128	256	128
Polymyxins	Polymyxin B	0.5 (S)	0.125 (S)	0.125 (S)	0.25 (S)	0.25 (S)	0.25 (S)	0.25 (S)
Tetracyclines	Doxycycline	8 (I)	1 (S)	1 (S)	2 (S)	4 (S)	2 (S)	0.5 (S)
	Tetracycline	128 (R)	128 (R)	32 (R)	128 (R)	64 (R)	128 (R)	32 (R)
	Tigecycline	1 (S)	0.25 (S)	0.25 (S)	1 (S)	0.25 (S)	1 (S)	0.25 (S)

^*a*^ Clinical antimicrobial susceptibility interpretive criteria are included in the brackets for β-lactams (carbapenems and third-generation cephalosporins), an aminoglycoside, fluoroquinolones, polymyxin, and tetracyclines (42, 43).

Overall, the disruption of one of the three RND pumps (AdeB, AdeG [AdeFGH] and AdeJ [AdeIJK]) rendered planktonic cells of the mutants less resistant to carbapenems, fluoroquinolones, amikacin, azithromycin, and tetracyclines. Specifically, the MICs of imipenem, amikacin, levofloxacin, doxycycline, azithromycin, and chloramphenicol were significantly decreased. Of the three efflux systems, the *adeB* inactivation reduced resistance (with 4-fold to 16-fold MIC decreases) to imipenem, meropenem, amikacin, levofloxacin, azithromycin, polymyxin B, chloramphenicol, doxycycline, tetracycline, and tigecycline (Table 1). The *adeFGH* deletion caused a 4-fold MIC reduction with imipenem, chloramphenicol, and doxycycline, whereas the *adeIJK* knockout produced a 4-fold to 16-fold MIC decrease with levofloxacin, chloramphenicol, doxycycline, tetracycline, and tigecycline (Table 1). Within the AdeRS-AdeABC system, the inactivation of the accessory protein-encoding gene *adeA* or the outer membrane protein-encoding gene *adeC* resulted in less profound MIC reductions than the effect of the pump gene *adeB* disruption. The deletion of *adeRS* resulted in moderate MIC reductions of multiple antimicrobials (Table 1), suggesting that *adeRS* positively regulates *adeABC* expression in the AYE strain.

The changes in MIC values of antimicrobials for various efflux-deficient strains were further examined by comparing them with the clinical breakpoints (42, 43). Intriguingly, the deletion of *adeB* or *adeA* led to the change of a category status from resistant to susceptible for amikacin, whereas all efflux gene-disrupted mutants possessed the change from intermediate to susceptible for doxycycline (Table 1), highlighting the clinical relevance of targeting efflux-mediated resistance (17).

### Antimicrobial susceptibility of biofilm cells

Antimicrobial susceptibility of biofilm cells of *A. baumannii* AYE and its six efflux system deletion mutants was also compared. As expected, biofilm cells showed much higher resistance to various antimicrobials as shown with the minimal biofilm inhibitory concentration (MBIC) values (Table 2), compared with the MIC results for planktonic cells (Table 1). However, disruption of any of the *adeB*, *adeG,* and *adeJ* genes was able to yield various increased antimicrobial susceptibilities of biofilm cells, suggesting the functional contribution of RND pumps to biofilm antimicrobial resistance. In particular, the *adeB* deletion led to increased susceptibility to levofloxacin (with 64-fold reduction in MBIC), azithromycin (128-fold decrease), and tigecycline (8-fold to 16-fold decrease) (Table 2). The role of *adeIJK* in biofilm antimicrobial resistance was found to be less than that of *adeB* but more significant than that of *adeFGH* (Table 2).

**TABLE 2:** Effects of inactivating RND efflux system genes on antimicrobial susceptibility in biofilm cells of *A. baumannii*

Antibacterial drug	Minimal biofilm inhibitory concentration (MBIC) in μg/mL						
	AYE	△*adeA*	△*adeB*	△*adeC*	△*adeRS*	△*adeFGH*	△*adeIJK*
Meropenem	8	8	4	8	4–8	8	4
Imipenem	8	2	4	4	4	4	4
Ceftazidime	˃ 1024	˃ 1024	˃ 1024	˃ 1024	˃ 1024	˃ 1024	˃ 1024
Ceftizoxime	˃ 1024	˃ 1024	˃ 1024	˃ 1024	˃ 1024	˃ 1024	˃ 1024
Ampicillin	˃ 1024	˃ 1024	˃ 1024	˃ 1024	˃ 1024	˃ 1024	˃ 1024
Cefoperazone-Sulbactam	512	512	256	512	256	256	128
Amikacin	256–512	128	128	256	128	128–256	256
Ciprofloxacin	256–512	128	64–128	256	64–128	256–512	256–512
Levofloxacin	512	128	8	128–256	128–256	512	256
Azithromycin	512	8	4	128	256	512	64
Chloramphenicol	>1024	1024	256	1024	516	>1024	516
Polymyxin B	1024	64–128	32–64	128–256	128	512	64
Doxycycline	16–32	16	8	16–32	8–16	16	16
Tetracycline	512–1024	256–512	256	512	256	512–1024	512–1024
Tigecycline	4	1	0.25–0.5	1–2	0.5	2	1

### Effect on bacterial motility

**Fig 1:**
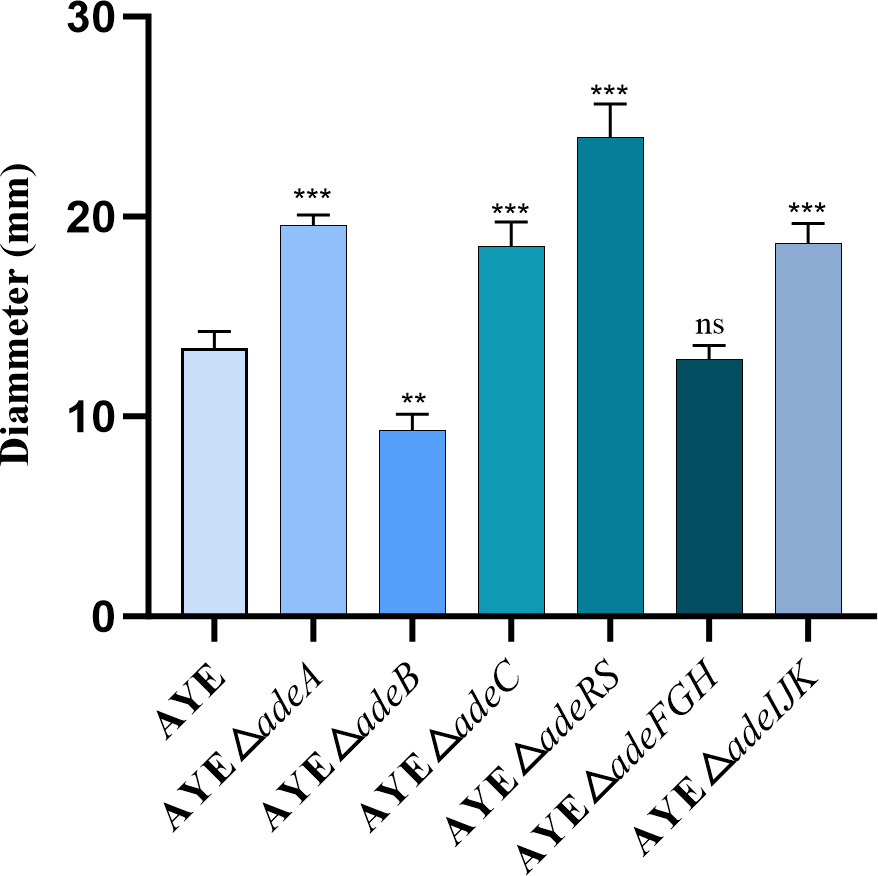
Surface motility ability of *A. baumannii* AYE and its six RND efflux system gene deletion mutant strains. Twitching motility comparison in the presence of NaCl (1%) ($\bar{x}$ ± s, *n* = 3; Compared with the parental strain AYE, ***, *P* < 0.001; **, *P* < 0.01; *, *P* < 0.05; ns [no statistical significance], *P* > 0.05).

Bacterial motility abilities were overall enhanced significantly after an efflux system gene deletion (except Δ*adeB* and Δ*adeFGH*) using a twitching motility assay (Fig. 1; Fig. S4). The deletion of *adeA*, a*deC*, *adeRS,* or *adeIJK* resulted in elevated motility (19–23 mm, with reduced mobility [9 mm] with Δ*adeB* and no motility change with Δ*adeFGH*), compared with that of the parental strain AYE (13 mm) (Fig. 1; Fig. S4). NaCl at 0.25%, 0.5%, and 1% showed no significant effect on the observed motility in this assay (Table S3). The △*adeRS* mutant exhibited the most significant enhancement in motility (i.e., that is 24 mm) (Fig. 1).

### Effect on bacterial adhesion and human serum susceptibility

**Fig 2:**
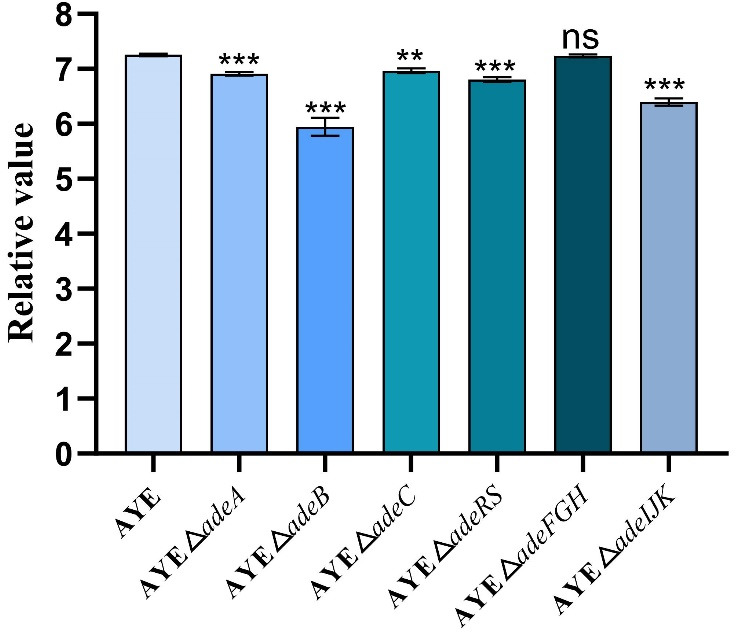
The adhesion abilities of *A. baumannii* AYE and its six RND efflux system deletion mutant strains ($\bar{x}$ ± s, *n* = 3; Compared with the parental strain AYE, ***, *P*＜0.001; **, *P*＜0.01; ns [no statistical significance], *P*＞0.05).

The relative value method (lg_10_ [*N* × 20 × 1,000]) (44, 45) was applied to determine the relative effect of the efflux system gene deletion on bacterial adhesion. As shown in Fig. 2, compared with the parental strain AYE (7.25 ± 0.03 [relative value]), the adhesion ability of the efflux gene deletion mutants decreased to varying degrees. Among them, the △*adeB* strain showed the most significant decrease in adhesion ability (5.95 ± 0.16), followed by strains △*adeIJK* (6.40 ± 0.07), △a*deRS* (6.81 ± 0.05), △*adeA* (6.91 ± 0.04), and △*adeC* (6.96 ± 0.05). Furthermore, the adhesion ability of strain △*adeFGH* (7.24 ± 0.03) showed no significant change. It is worth mentioning that we used six-well plates without tissue culture treatment. The cell culture dish materials usually have low chemisorption, and thus, the lack of tissue culture treatment may reduce the adsorption of bacterial cells to the plates.

From the serum-killing assay, the parental strain and all deletion mutants showed no significant differences in susceptibility to human serum (complement) (Fig. S6). This result was consistent with the findings in the literature not showing the efflux pump inactivation with altered serum susceptibility.

### Effect on bacterial biofilm formation

**Fig 3:**
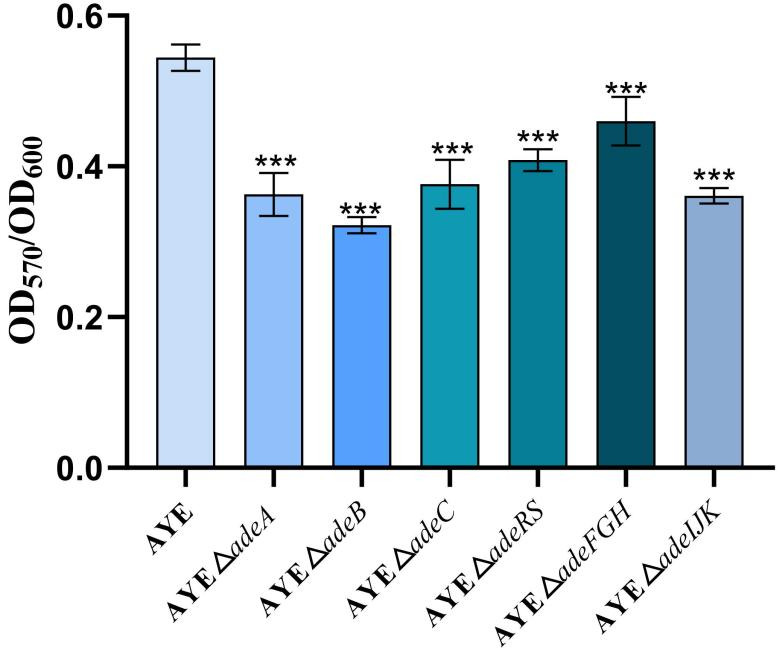
Bacterial biofilm formation ability of *A. baumannii* AYE and its six efflux system gene deletion mutant strains ($\bar{x}$ ± s, *n* = 5; Compared with the parental strain AYE, ***, *P*＜0.001).

*A. baumannii* AYE and its RND efflux gene deletion strains were able to form biofilm. However, the biofilm formation quantities of the gene deletion mutant strains decreased to varying degrees when compared with the parental strain AYE (Fig. 3; Table S4). Among them, strain *△adeB* exhibited the most significant reduction in biofilm formation (41% reduction), followed by strains *△adeIJK* (34%), *△adeA* (33%), *△adeC* (31%), *△adeRS* (25%), and *△adeFGH* (16%). Compared with the biofilm formation of the AYE strain, these reductions in biofilm quantities of the gene deletion strains were statistically significant (*P* < 0.001).

### Virulence of RND efflux system deletion mutant strains in a *Galleria mellonella* infection model

**Fig 4:**
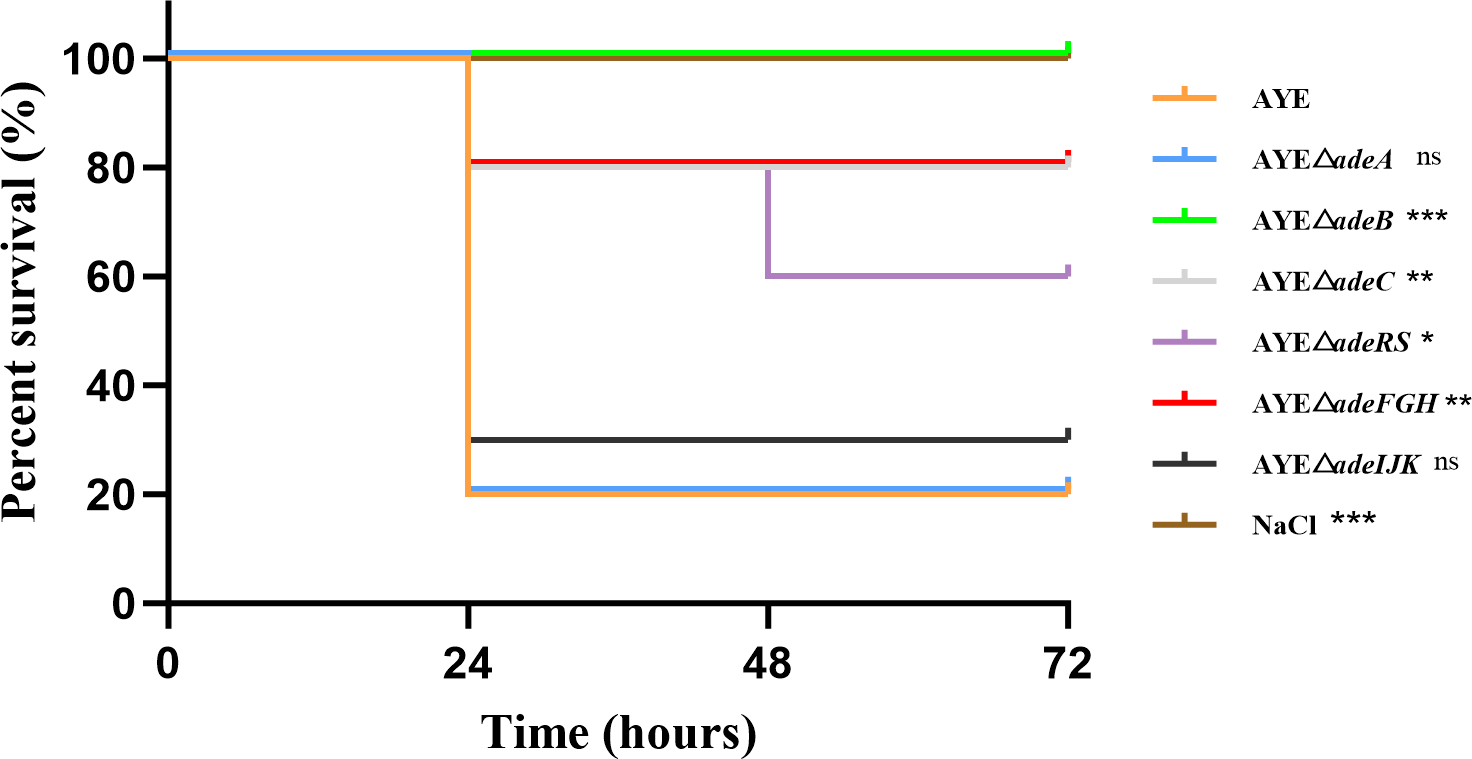
Kaplan-Meier survival curve (%) showing the virulence of individual isogenic strains of *A. baumannii* AYE and its six RND efflux system deletion mutants to *G. mellonella* (*n* = 10 per group; Compared with the parental strain AYE: ***, *P*＜0.001; **, *P*＜0.01; ns [no statistical significance], *P*＞0.05). The experiment was conducted three times.

Bacterial virulence conferred by *A. baumannii* AYE and its efflux system disrupted strains was measured by examining the survival rate of *G. mellonella* (caterpillars of the greater wax moth) after administering with the pathogen. As illustrated in Fig. 4, after 72 h post-treatment with strain AYE infection, the survival rate of *G. mellonella* was 20%. However, following infection with gene knockout strains, the survival rates were as follows: strain △*adeA*, 20% (no change compared with the parent strain); △*adeIJK*, 30%; △*adeRS*, 60%; △*adeFGH* and △*adeC*, 80%; whereas strain △*adeB* exhibited a *G. mellonella* survival rate of 100% at 72 h post-infection.

### Expression levels of virulence factor-related genes in RND efflux system gene deletion strains

The role of the AdeABC, AdeFGH, and AdeIJK efflux systems in bacterial virulence or pathogenicity-related functions was assessed by determining the impact of the efflux system gene deletion on the expression of virulence/pathogenicity genes in *A. baumannii*. For the latter, a total of seven genes were targeted for comparing their expression profiles via the RT-qPCR assay with primers included in Table S2. The seven genes include: (i) *abaI* encoding a quorum sensing system auto-inducer synthase (46); (ii) *abaR* encoding a quorum sensing system auto-inducer synthase receptor (46); (iii) *bap* encoding biofilm-associated protein Bap (47); (iv) *bfmR* encoding response regulator BfmR of BfmRS two-component system (48); (v) *csuE* encoding adhesin CsuE of the Csu pilus subunits CsuA/B/C/E (49); (vi) *ompA* encoding outer membrane protein OmpA (50, 51); and (vii) *pgaA* encoding outer membrane protein PgaA (52).

**Fig 5:**
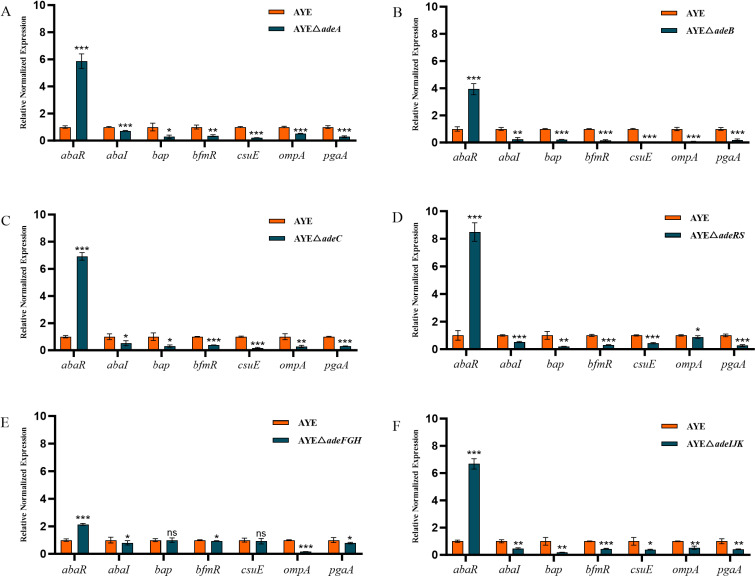
Relative expression levels (in 2^−ΔΔCT^) of virulence factor-related genes in *A. baumannii* AYE and its six RND efflux system gene deletion mutant strains (A. Δ*adeA*; B. Δ*adeB;* C. Δ*adeC;* D. Δ*adeRS*; E. Δ*adeFGH*; F. Δ*adeIJK*) ($\bar{x}$ ±s, *n* = 3; Compared with the parental strain AYE: ***, *P* ＜0.001; **, *P* ＜0.01; *, *P* < 0.05; ns [no statistical significance], *P*＞0.05).

As shown in Fig. 5; Table S5, the expression levels of the *abaR* gene increased to varying degrees in six gene deletion strains. Among them, the increase was most significant in strain AYE△*adeRS*, with the expression level upregulated approximately 8.5 times compared with strain AYE. However, with the disruption of those efflux system genes, the expression levels of *abaI*, *bap*, *bfmR*, *csuE*, *ompA,* and *pgaA* exhibited a downward trend. Among them, the downregulation was most pronounced in strain AYE△*adeB*, with *abaI*, *bap*, *bfmR*, *csuE*, *ompA,* and *pgaA* genes downregulated approximately 4.2, 4.8, 6.7, 33.3, 16.7, and 5.3 times, respectively.

## DISCUSSION

Multidrug efflux systems in gram-negative pathogens such as *A. baumannii* play a critical role in both intrinsic and acquired multidrug resistance and also serve other functions including biofilm formation and virulence factors (3, 4, 17, 53, 54). This study took an approach to simultaneously compare the role of the three RND pumps, AdeABC, AdeFGH, and AdeIJK, in impacting antimicrobial resistance and virulence factor production in a multidrug-resistant isolate, *A. baumannii* AYE. With its complete genome sequence reported as early as 2006 (10), *A. baumannii* AYE provides a great advantage to particularly understanding the efflux contribution in the presence of multiple antimicrobial-specific resistance mechanisms.

In addition to the presence of RND pumps, *A. baumannii* AYE possesses a range of antimicrobial class-specific resistance mechanisms, including the production of three β-lactamases (Ambler classes A, C, and D enzymes) and nine aminoglycoside-modifying enzymes, the presence of two phenicol-specific efflux pumps and three tetracycline-specific efflux pumps, and the fluoroquinolone gyrase target alteration (Table S6) (10). In this regard, various resistance mechanisms are expected to interplay to raise bacterial resistance levels, including the synergistic effect between RND pumps and other resistance mechanisms (55). However, there is a lack of simultaneous comparison of AdeABC, AdeFGH, and AdeIJK in sequenced multidrug-resistant *A. baumannii*. A previous study used a susceptible *A. baumannii* with laboratory-generated efflux overproducers to compare the contribution of RND pumps with resistance and biofilm formation (26). In our study, we aimed to understand how RND pumps could play their functional roles in the presence of high-level multidrug resistance. First, the three tripartite RND efflux systems in multidrug-resistant *A. baumannii* AYE were found to be expressed constitutively. In this regard, *adeIJK* is known to express constitutively in susceptible isolates to mediate intrinsic resistance and to be overproduced in resistant isolates to provide acquired resistance (4, 17, 24). *adeABC* may be expressed and provide no or only moderate intrinsic resistance, but when overproduced in resistant isolates, it mediates acquired resistance (4, 17, 21, 22). When expressed in resistant isolates, *adeFGH* contributes to acquired resistance with narrow substrate profiles such as chloramphenicol (4, 23). The higher expression of *adeC* than that of *adeAB* of the *adeABC* operon in this study aligns with the *adeABC* and *adeC* transcripts identified in a Northern hybridization assay (28). Subsequently, in our investigation, inactivation of one of the RND pumps or their components led to reduced resistance to multiple antimicrobials of various classes, and this observation is particularly evident with the AdeB pump inactivation (Table 1), highlighting that *Acinetobacter* RND pumps likely function synergistically with other antimicrobial-specific resistance. For example, in the presence of several aminoglycoside-modifying enzymes and major facilitator superfamily tetracycline efflux transporters in the AYE strain (Table S6), the *adeB* gene deletion was still able to reduce resistance to amikacin and three tetracyclines tested (Table 1). However, amikacin and tetracycline MIC values (16 and 32 µg/mL, respectively) for the *adeB* mutant are still relatively high, consistent with the co-presence of other resistance mechanisms (such as those listed in Table S6). Moreover, with the production of predominant β-lactamases (i.e., extended-spectrum β-lactamase VEB-1, AmpC β-lactamase ADC-11, and oxacillin-hydrolyzing β-lactamase OXA-10), efflux contribution to third-generation cephalosporins cannot be uncovered due to high MIC values of >1024 µg/mL (Table 1). Nonetheless, the β-lactam-β-lactamase inhibitor combination, cefoperazone-sulbactam, allows us to observe the contribution of AdeABC to resistance to third-generation cephalosporin (cefoperazone)-β-lactamase inhibitor (sulbactam).

By comparing antimicrobial susceptibility changes of the RND system mutants, AdeABC is revealed to be likely more potent than AdeFGH or AdeIJK in the AYE strain. In particular, the contribution of AdeFGH to resistance appears to be limited in the AYE strain with a moderate effect on reduced resistance, and this result could be due to the presence of AdeABC that possibly masks the roles of other efflux systems. Indeed, an investigation of AdeFGH was carried out by inactivating AdeABC and AdeIJK efflux systems (23). Inactivation of AdeFGH triggered the overproduction of AdeABC in a susceptible *A. baumannii* strain (56). Moreover, for the AdeABC system, the effect from *adeA* or *adeC* disruption is less profound than that of the *adeB* pump gene deletion, which is consistent with the observation that other homologs of accessory proteins or outer membrane proteins of the RND efflux systems can function with an RND transporter (which determines the pump substrate specificity) as observed in multiple gram-negative bacteria including *A. baumannii*, *E. coli,* and *P. aeruginosa* (17). Thus, the impact from the deletion of a transporter gene (e.g., *adeB*) can be equivalent to that of the deletion of the entire three-gene operon (e.g., *adeABC*). Finally, our findings also expand the current understanding of Ade pump substrate profiles to include azithromycin, doxycycline, levofloxacin, and polymyxin B (22–26, 28).

Our study also included the inactivation of the AdeRS regulatory system that regulates AdeABC. Here, it is necessary to highlight another study by Richmond et al. (35) that examined the impact of the inactivation of AdeRS and AdeB on multidrug resistance, biofilm formation, and virulence of two *A. baumannii* strains (including strain AYE), providing a direct comparison of the findings from our study. With the RNA-seq approach, Richmond et al. (35) uncovered that loss of AdeRS (or AdeB) significantly altered the expression of many genes, particularly those associated with antimicrobial resistance and virulence interactions. Inactivation of AdeRS in strain AYE from Richmond et al. (35) and us in this study showed reduced resistance, supporting that AdeRS positively regulates AdeABC (28).

The relation between RND pumps and biofilm resistance/formation has drawn much attention but remains an area for further research (17, 26). Our findings support the contribution of RND Ade pumps to the resistance of biofilm cells, which aligns with the molecular basis of RND pumps in conferring resistance of planktonic cells. Regardless of molecular mechanisms of resistance, bacterial biofilm cells are known to generally exhibit much higher resistance than their planktonic cells in various bacteria (57, 58), including *A. baumannii* isolates (59). Our study indicates that RND pump inactivation can reduce biofilm antimicrobial resistance levels.

Biofilm formation is part of bacterial virulence and pathogenicity. In *A. baumannii*, inactivation and overproduction of an Ade efflux system could both negatively affect biofilm formation; for example, the *adeB* deletion mutant and overproducer had significant defects of 39% and 59%, respectively, in biofilm formation (26), suggesting that a balanced pump expression would be needed for optimal biofilm production. Our findings also revealed that Ade pumps are involved in biofilm formation, with AdeABC being relatively more potent, as the results with the *adeB* deletion show the most significant reduction of biofilm cells (at 41%). Tripartite RND pumps are typically encoded by an operon, and thus, the deletion of one of the operon genes (particularly the transporter-encoding genes such as *adeB*) could disrupt or affect the pump function related to resistance and non-resistance functions (17). RND pumps are described to possess at least four roles in biofilm formation, including (i) efflux of extracellular polymeric substances and/or quorum sensing/quenching molecules; (ii) indirect regulation of genes involved in biofilm formation; (iii) efflux of harmful molecules; and (iv) influencing aggregation through promoting or preventing adhesion to surfaces and other cells (60). Bacterial motility as a virulence factor was reported in *A. baumannii* (34, 35, 61). Bacterial surface-associated motilities of RND pump mutants are generally increased, with the strongest effect occurring in the *adeRS* deletion mutants (Fig. 1). The latter observation for the effect from the *adeRS* deletion is consistent with the elevated expression of genes encoding motility such as competence *com* genes (35). The motility alterations could help planktonic cells move close to the surface in the early stage of biofilm formation (62). Intriguingly, the twitching assay revealed no effect from three NaCl concentrations tested on bacterial motility (Table S4). However, we observed that the decreasing NaCl concentrations (0.125% and 0.25% from 0.5%) in the tryptone soft agar media led to higher surface-associated mobility in both the parental strain and its pump mutants (Fig. S5), similar to that previously reported (61), suggesting a potential role of RND systems in response to salinity stress. Still, it is important to note that *Acinetobacter* species generally lack flagella, thus not swimming, and additional motility tests are warranted to help understand the motility alterations of the RND system mutants (34, 61). Furthermore, the reduction of the adhesion abilities of the RND efflux mutants (except Δ*adeFGH*) also correlates well with the biofilm formation abilities as exemplified with the *adeB* mutant (Fig. 2 and 3). Thus, the adhesion change likely compromises biofilm formation. The latter itself is complex, involving numerous players and steps (49, 63). As well, we noted that some deletion mutants had delays in the early log growth phase but were able to have similar duplication times afterward. The early growth lag might also contribute to effects on the adhesion and biofilm production, although normalized OD_570_/OD_600_ ratios were used for measuring biofilm formation. It is worth further mentioning the biofilm formation results obtained by Richmond et al. (35) and us assessing the AYE isolate. Although both studies observed strong biofilm reduction in *adeB* deletion mutants, we were able to also note the reduced biofilm formation on plastic due to the *adeRS* deletion, but Richmond et al. (35) indicated the biofilm formation reduction of their *adeRS* mutant only in an *ex vivo* porcine mucosal tissue but not on plastic. However, although there was no statistically significant difference, we noted the varied, wide-range biofilm formation alterations of the *adeRS* mutant on plastic (i.e., reduced and increased when compared with that of the parental AYE strain) in that study (35).

The reduced virulence phenotype of some RND pump mutant strains (except Δ*adeA* and Δ*adeIJK*) is evident with the elevated survival rates (i.e.*,* reduced mortalities) of *G. mellonella* larvae administered with *A. baumannii* efflux pump mutants. *G. mellonella* provides a nonmammalian model system of infection to assess host-pathogen interactions, particularly for the pathogenesis of gram-negative bacteria, including *A. baumannii* (64–66). Intriguingly, Richmond et al. (35) reported the attenuated virulence of the *adeB* deletion mutant as confirmed by us in this study, but no changes with the *adeRS* deletion mutant. However, we observed the effect of the *adeRS* deletion in reducing the mortality of *G. mellonella*, which is predicted by the regulatory role of AdeRS on AdeABC. The virulence attenuation observed in the *G. mellonella* model is further compared with *A. baumannii* virulence gene expression. Determination of bacterial virulence factors has been challenging but greatly improved in recent years with advances in genome sequencing technologies (67). In this regard, several virulence factor-related genes tested in this study (i.e., *abaI, abaR*, *bap*, *bfmR*, *csuE*, *ompA*, and *pgaA*; Table S2; Fig. 5) are part of genetic determinants encoding products that contribute to quorum sensing, bacterial adhesion, and biofilm formation. These well-characterized virulence factor genes can serve as major biomarkers to assess *A. baumannii* virulence and pathogenicity. As such, the findings with the downregulated *abaI* and upregulated *abaR* are consistent with the role of the AbaI/AbaR system in quorum sensing regarding virulence phenotype (68). Biofilm-associated protein Bap is needed for mature biofilm formation (47, 69). Adhesin CsuE is part of the chaperone (CsuC)-adhesin (CsuE) preassembly complex that forms the basis for bacterial attachment to abiotic surfaces (49). Outer membrane surface protein PgaA is encoded by the *pgaABCD* locus that confers the production of poly-β−1–6-*N*-acetylglucosamine, a molecule critical for biofilm formation (52). The decreased *pgaA* expression noted in this study aligns with the *pgaC* expression reduction using RNA-Seq assay (35). Finally, OmpA is an outer membrane protein constituting a major risk factor related to *A. baumannii* infection (50, 51). Together, reduced expression of various virulence factors provides the molecular basis to understand the observations on the decreases of biofilm formation and virulence effect from RND pump inactivation, highlighting the RND pumps as potential therapeutic targets against *A. baumannii* infection. Further research is warranted to particularly elucidate interplays among multiple RND pumps themselves and between these RND pumps and other mechanisms related to resistance and virulence.

## MATERIALS AND METHODS

### Bacterial strains, growth media, and antimicrobial agents

*A. baumannii* AYE is a multidrug-resistant strain (10, 40), and its six deletion mutants (i.e., Δ*adeA*, Δ*adeB*, Δ*adeC*, Δ*adeRS*, Δ*adeFGH*, and Δ*adeIJK*) were constructed in this study. *A. baumannii* ATCC 17898 *and* ATCC 19606 were used as susceptible reference strains (40, 70, 71). *E. coli* ATCC 25922 is a quality control strain for antimicrobial susceptibility testing (42). *E. coli* DH5α is a laboratory strain used in cloning for recovering recombinant plasmids. *E. coli* S17-1 (ATCC 47055) is used for mobilizing plasmids into *A. baumannii* AYE.

Bacterial cells were cultured in Luria Bertani (LB) broth medium (1% tryptone, 0.5% yeast extract, and 1% sodium chloride), LB agar medium, tryptone soy broth (TSB) medium (1.7% tryptone, 0.3% soya peptone, 0.5% sodium chloride, 0.25% glucose, and 0.25% dipotassium phosphate), or cation-adjusted Muller-Hinton broth (CAMHB) (containing 0.3% beef extract, 1.75% casein acid hydrolysate, 0.15% starch, and 0.005% calcium chloride) as described in relevant experiments. These media were purchased from Haibo Biotechnology (Qingdao, China). Clinically relevant antimicrobial agents used for antimicrobial susceptibility testing include β-lactams (meropenem, imipenem, cefotaxime, ceftazidime, ampicillin, and cefoperazone-sulbactam), aminoglycoside (amikacin), fluoroquinolones (ciprofloxacin and levofloxacin), macrolide (azithromycin), phenicol (chloramphenicol), polymyxin (polymyxin B), and tetracyclines (doxycycline, tetracycline and tigecycline) that were all purchased from Meilun Biological (Dalian, China).

### Gene expression assay

RT-qPCR was used to assess the gene expression of relevant RND efflux pump/regulator genes (i.e., *adeA*, *adeB*, *adeC*, *adeR*, *adeS*, *adeG,* and *adeJ*) and virulence factor or biofilm formation genes (*abaI, abaR*, *bfmR*, *csuE*, *bap*, *pgaA,* and *ompA*) of *A. baumannii* strains. The primers used for the target genes and their encoding products are presented in Table S1. Briefly, total RNA was prepared using an RNA isolation kit (Vazyme Biological, Nanjing, China) including DNase treatment from *A. baumannii* AYE and its six gene deletion derivative mutants, followed by RT-qPCR that included the reverse transcription of RNA into cDNA using a cDNA synthesis kit (Vazyme) and qPCR using the fluorescent dye SYBR Color qPCR Master Mix (Vazyme). PCR alone without reverse transcription was included to confirm the absence of DNA contamination. The qPCR cycling conditions were as follows: pre-denaturation at 95°C for 3 min, followed by 40 cycles of 95°C for 10 s and 60°C for 30 s. The 16S rRNA gene was used as the reference gene, whereas the target genes included the efflux system *ade* genes or virulence genes. Strain AYE served as the control, and the experimental groups included the relevant efflux system gene deletion mutants or two ATCC strains. The expression level of each gene was normalized, and the relative expression was calculated as 2^−ΔΔCT^. For comparing the efflux pump gene expression in one strain (*A. baumannii* AYE, ATCC 17978 and ATCC 19606), the *adeA* expression was used for comparing with the expression of another *ade* gene.

### Construction of RND efflux system deletion mutants

RND efflux system component genes (*adeA, adeB*, *adeC*, *adeRS*, *adeFGH,* and *adeIJK*) of *A. baumannii* AYE strain were targeted for constructing relevant unmarked in-frame gene deletion mutants using the marker-free gene deletion method (72). Briefly, PCRs were performed to amplify sequences upstream and downstream from each of those target genes using the *A. baumannii* AYE genomic DNA as the template. Primers used for the gene deletions and their confirmation were designed based on the genome sequences of *A. baumannii* AYE and are listed in Table S2. These primers were synthesized by Sangon Biotech (Shanghai, China). For the deletion of *adeA, adeB*, *adeC,* and *adeRS*, the upstream PCR fragments of relevant target genes carried the *Not*I site, while the downstream PCR fragments contained the *Bam*HI site. A fusion PCR method was used to amplify the fused fragment using the upstream and downstream PCR products as the templates, followed by PCR product purification and double digestion with *Not*I and *Bam*HI enzymes. For the deletion of *adeFGH* and *adeIJK*, the upstream PCR fragments carried *Not*I and *Bam*HI sites, whereas the downstream PCR fragments contained *Bam*HI and *Sph*I sites. These upstream and downstream fragments were digested with *Not*I-*Bam*HI and *Bam*HI-*Sph*I, respectively. Subsequently, relevant restriction enzyme-treated PCR fragments were cloned into the suicide vector pMo130-Tel^R^ (pretreated with the relevant restriction enzymes), followed by the transformation of chemically generated competent *E. coli* DH5α cells. The latter were plated and then selected on LB agar plates containing kanamycin at 50 µg/mL after overnight incubation at 37°C. Relevant gene deletion constructs (i.e., the suicide vector carrying the homologous fragments with the relevant target deletion) were obtained from *E. coli* transformants, followed by plasmid isolation, PCR verification, and DNA sequencing. The deletion constructs were further transformed through electroporation into an electrocompetent host strain of *E. coli* S17-1 with the selection on kanamycin-containing LB agar plates.

The resultant plasmids from *E. coli* S17-1 were mobilized into *A. baumannii* AYE via biparental conjugation. Briefly, overnight LB cultures of *E. coli* S17-1 carrying a deletion construct (donor) and *A. baumannii* AYE (recipient) were prepared. In a 2 mL Eppendorf tube, the donor and recipient cells were mixed in a ratio of 3:1, followed by centrifugation, washing, and resuspending in saline. The bacterial suspension was loaded on a nitrocellulose membrane (0.45 µm pore size) and then placed on LB agar for incubation at 37°C for 6–8 h. Bacterial cells on the nitrocellulose membrane were resuspended, centrifuged, and washed in LB broth, followed by resuspending in saline. Biparental cells were then plated on LB agar with potassium tellurite (30 µg/mL) and gentamicin (16 µg/mL) and incubated at 37°C for 16–20 h. Transconjugants carrying relevant deletion genes in the chromosome obtained after the first cross-over were selected for the inheritance of tellurite resistance and *xylE*^+^ (yellow colonies). During the second cross-over, mutants with gene deletion were selected for loss of *sacB* by passing the first cross-over recombinants in LB agar containing 10% sucrose. Sucrose-resistant colonies arising after overnight incubation at 37°C were screened for the presence of the target gene deletion by using PCR, followed by sequencing confirmation (Fig. S1 and S2).

### Planktonic cell antimicrobial susceptibility assay

The MIC values of 15 antimicrobial agents for *A. baumannii* strains were measured by a 2-fold serial broth dilution method using CAMHB. Briefly, *A. baumannii* strains were inoculated on LB agar medium at 37°C for 16–20 h, then resuspended in saline (0.9% sodium chloride) to produce a bacterial suspension of OD_600_ = 0.10, and diluted 20 times for use. Antimicrobial stock solutions were prepared each at a concentration of 10,240 µg/mL using an appropriate solvent and were further made via a 2-fold serial dilution as needed. To 180 µL of CAMHB in each well of a 96-well plate, 10 µL of bacterial suspension and 10 µL of the appropriate antimicrobial solutions were added, respectively. The plates were incubated at 37°C for 20 to 24 h, and then, the bacterial growth was measured using a Full Wavelength Microplate Reader (BioTeck). The values of ≤OD_600_ of 0.1 were determined as no bacterial growth.

### Biofilm cell antimicrobial susceptibility assay

This assay was conducted based on the reference of Kart et al. (73), with modifications. Briefly, 20 µL of bacterial cell suspension with OD_600_ = 0.10 and 180 µL of TSB were inoculated into 96-well plates, followed by incubation at 37°C for 24 h to form biofilm. Subsequently, the culture medium was aspirated, and each well was gently washed three times with 200 µL of phosphate-buffered saline. Finally, 190 µL of CAMHB medium was added to each well along with 10 µL of the corresponding concentration of the antimicrobial solution. The plates were then incubated at 37°C for 20–24 h, and the wells with OD_600_ values of less than 0.10 were considered no growth for MBIC results.

### Bacterial motility ability assay

Bacterial surface-associated motility was examined using the twitching motility as described by Antunes et al (34). Briefly, LB medium plates (1% tryptone, 0.5% yeast extract, 0.25%, 0.5% or 1% NaCl, and 0.5% agarose) were prepared, and bacterial cells (adjusted to OD_600_ = 0.10 from overnight culture) were inoculated with a pipette tip to the bottom of the polystyrene Petri dish. The plates were tightly closed with parafilm to prevent drying and incubated at 37°C for 24 h. Twitching motility was determined by removing the agarose layer, staining the plates with a 0.1% crystal violet solution for 30 min, and measuring the diameter of the bacterial motility.

### Bacterial adhesion assay

The adhesion ability of *A. baumannii* strains was assessed using the plate counting method. The strains were inoculated in LB broth and incubated at 37°C with shaking overnight. The bacterial culture corresponding to OD_600_ = 0.10 was diluted 1:1000. Then, 1 mL of the diluted bacterial suspension was added to six-well polypropylene culture plates without tissue culture treatment. The cultures were then incubated at 37°C for 4 h, washed three times with phosphate-buffered saline solution, followed by sonication for 10 min to dislodge the adhered bacteria. The suspension was diluted 20 times, and 100 µL of the dilution was spread on LB agar plates. The plates were then incubated at 37°C for 24 h before colony counting. The relative value method (lg10 [*N* × 20 × 1,000]) (44, 45) was used to assess the effect on bacterial adhesion of AYE strain and its efflux system deletion mutants.

### Susceptibility to normal human serum

Overnight cultures of *A. baumannii* cells were prepared to bacterial suspensions at a 0.5 McFarland standard density. Subsequently, 100 µL of bacterial cells (1 × 10^5^ CFU) and 900 µL of human serum (without or with heat-inactivation [56°C for 30 min]) were mixed and cultivated for one hour with shaking at 37°C, followed by plating various dilutions of the serum-treated cells on LB agar plates for bacterial cell counting (CFUs/mL).

### Bacterial biofilm formation determination

The crystal violet staining method was used to examine the biofilm formation ability of *A. baumannii* strains. Briefly, 180 µL of TSB medium was sequentially added into each well of a 96-well cell culture plate, followed by the inoculation of 20 µL of bacterial cells (with OD_600_ = 0.1) with 200 µL of TSB as the blank control wells. The plates were incubated at 37°C for 24 h, and then, OD_600_ values were measured using a Full Wavelength Microplate Reader (BioTek). Then, the wells were washed three times with phosphate-buffered saline to remove planktonic cells, and the biofilm cells were fixed with 200 µL of methanol for 20 min with the liquid being aspirated. 200 µL of 0.5% crystal violet was added to each well for staining for 15 min, and then, the crystal violet was gently aspirated. Later, the wells were washed three times with phosphate-buffered saline, followed by air dry for 20 min. Subsequently, 200 µL of 95% ethanol was added to each well and let sit for 15 min to dissolve the crystal violet completely, and then OD_570_ values were measured. Biofilm formation was determined as OD_570_/OD_600_.

### *G. mellonella* infection experiment

*G. mellonella* serves as a model system to examine *A. baumannii* pathogenesis (64). Single colonies of *A. baumannii* AYE and its RND pump deletion mutants were inoculated into LB broth overnight (16–18 h) at 37°C. Bacterial cells were harvested by centrifugation and washed with sterile phosphate-buffered saline. Then, the bacterial suspension was prepared to a 0.5 McFarland standard density and was further diluted by 10-fold. *G. mellonella* larvae with a length of approximately 15–25 mm and a weight ranging from 250 to 350 mg were selected for the experiment. These *G. mellonella* had a pale-yellow color with no gray markings on their body surface. Ten larvae per group were randomly allocated; 20 µL of bacterial suspension was injected into the body of the second-to-last left/right hind leg of *G. mellonella* larvae. The no-treatment blank control group was administered with saline. The treated larvae were reared in a constant temperature incubator set at 37°C. The mortality of *G. mellonella* larvae was recorded every 24 h post-treatment for 72 h.

### Statistical analysis

The means and their standard deviations were calculated using SPSS 27.0. GraphPad Prism 10.0 was used for mapping. Data were analyzed with a one-way analysis of variance (ANOVA) followed by Dunnett’s test. Data were considered statistically significant with *P* < 0.05.

## Data Availability

The original contributions presented in this study are included in the article; further inquiries can be directed to the corresponding authors.

## SUPPLEMENTAL MATERIAL

Supplemental material aac.01858-24-s0001.docx Fig. S1 to S6; Tables S1 to S6.

ASM does not own the copyrights to Supplemental Material that may be linked to, or accessed through, an article. The authors have granted ASM a non-exclusive, world-wide license to publish the Supplemental Material files. Please contact the corresponding author directly for reuse.
