# Supplementary material for: Contribution of RND superfamily multidrug efflux pumps AdeABC, AdeFGH, and AdeIJK to antimicrobial resistance and virulence factors in multidrug-resistant Acinetobacter baumannii AYE
Source: Antimicrob Agents Chemother. 2025 May 23;69(7):e01858-24. doi: 10.1128/aac.01858-24 (PMC12217454; doi:10.1128/aac.01858-24)
Supplement: Supplemental material — Fig. S1 to S6; Tables S1 to S6. [file aac.01858-24-s0001.docx]

**Contribution of RND Superfamily Multidrug Efflux Pumps AdeABC, AdeFGH and AdeIJK to Antimicrobial Resistance and Virulence Factors in Multidrug-Resistant *Acinetobacter baumannii* AYE**

**Supplemental Materials**

**Fig S1.** Relative expression (in 2^−ΔΔCT^) of RND efflux system genes in *A. baumannii* AYE. A. Comparison of the expression of RND efflux system genes among strains AYE, ATCC 17978 and ATCC 19606. The gene expression of strain AYE is used for comparing with that of the corresponding genes of two ATCC strains. B. Expression of RND efflux system genes (pump genes *adeB*, *adeG* and *adeJ*; accessory membrane fusion protein gene *adeA*, outer membrane protein gene *adeC* and two-component regulatory system genes *adeRS*) in strain AYE. Because of the one strain used, the gene expression in 2^−ΔΔCT^ is relative to the *adeA* expression. ($\bar{x}$ ± s, n=3; Compared to strain AYE, ***, *P* < 0.001; **, *P* < 0.01; *, *P* < 0.05; ns [no statistical significance], *P*＞0.05).

**Fig S2.** PCR confirmation of AYE△*adeA*, AYE△*adeB*, AYE△*adeC* and AYE△*adeRS* mutants by agarose gel electrophoresis.

**Fig S3.** PCR confirmation of AYE△*adeFGH* and AYE△*adeIJK* mutants by agarose gel electrophoresis.

**Fig S4.** Twitching motility comparison of *A. baumannii* AYE and its six RND efflux system gene deletion mutants on soft agarose plates in the presence of 1% NaCl. The results are representative of three independent experiments showing similar results.

**Fig S5.** Swarming motility comparison of *A. baumannii* AYE and its six RND efflux system gene deletion mutants on soft agar plates and the effect of NaCl (0.5% [A], 0.25% [B] and 0.125% [C]). The results are representative of three independent experiments showing similar results.

**Fig S6.** Susceptibility of *A. baumannii* AYE and its six RND efflux system gene deletion mutant strains to normal human serum.

**Table S1.** Target genes and the primer sequences used for gene expression experiments.

**Table S2.** Primers used in the construction of the RND efflux system gene deletion mutants of *A. baumannii*.

**Table S3.** Twitching motility of *A. baumannii* AYE and its six RND efflux system gene deletion mutant strains as well as the effect of NaCl.

**Table S4.** Biofilm formation ability of *A. baumannii* AYE and its six RND efflux system gene deletion mutant strains.

**Table S5.** Expression levels of virulence factor-related genes in *A. baumannii* AYE and its six RND efflux system gene deletion mutant strains.

**Table S6.** Antimicrobial specific resistance determinants of *A. baumannii* AYE based on the complete genome sequence.

**Fig S1.** Relative expression (in 2^−ΔΔCT^) of RND efflux system genes in *A. baumannii* AYE. A. Comparison of the expression of RND efflux system genes among strains AYE, ATCC 17978 and ATCC 19606. The gene expression of strain AYE is used for comparing with that of the corresponding genes of two ATCC strains. B. Expression of RND efflux system genes (pump genes *adeB*, *adeG* and *adeJ*; accessory membrane fusion protein gene *adeA*, outer membrane protein gene *adeC* and two-component regulatory system genes *adeRS*) in strain AYE. Because of the one strain used, the gene expression in 2^−ΔΔCT^ is relative to the *adeA* expression. ($\bar{x}$ ± s, n=3; Compared to strain AYE, ***, *P* < 0.001; **, *P* < 0.01; *, *P* < 0.05; ns [no statistical significance], *P*＞0.05).

**
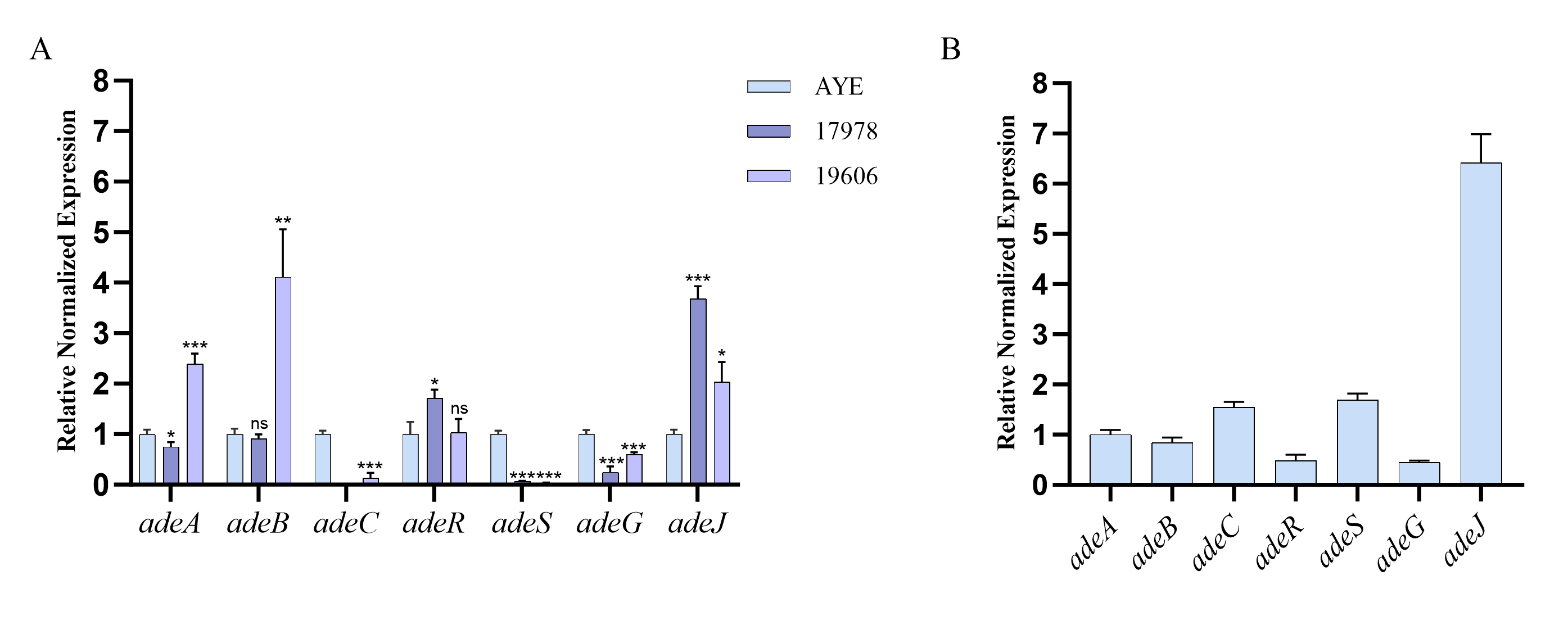
**

**Fig S2.** PCR confirmation of AYE△*adeA* (bottom left panel)*,* AYE△*adeB* (top left)*,* AYE△*adeC* (bottom right) and AYE△*adeRS* (top right) mutants by agarose gel electrophoresis.

Lane M: DNA size markers. Lanes 1 in all four panels used upstream/downstream primers specific to the genes in question (i.e., *adeA*, *adeB*, *adeC* and *adeRS*) with PCR products to smaller in the deletion mutants than those from the parental strain AYE; Lanes 2, 3 and 4 used, respectively, primer pairs AYE plasmid p2 F/R, AYE plasmid p4 F/R, and AYE csy F/R to amplify PCR products that ensured bacterial cells as *A. baumannii* AYE and its derivatives. Lanes 5 used primers p130-F/R with no PCR products detected to ensure that the pMo130-Tel^R^ vector was eliminated from the chromosome after the second crossover on the selection plate.


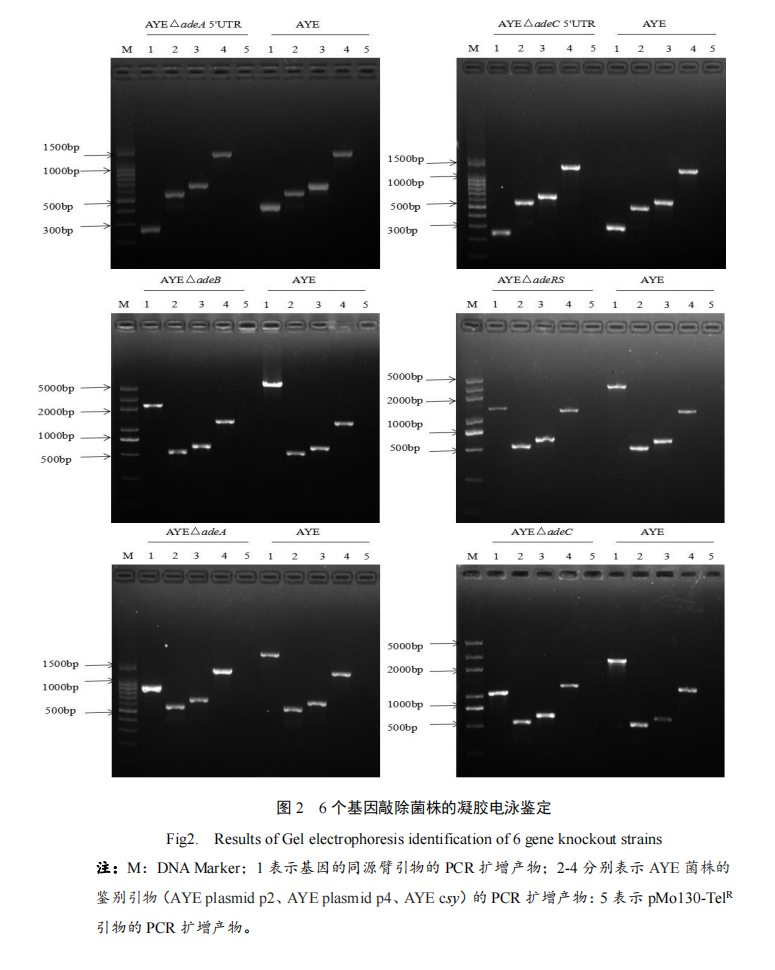


**Fig S3.** PCR confirmation of AYE△*adeFGH* and AYE△*adeIJK* mutants by agarose gel electrophoresis.

Lane M: DNA size markers; Lanes 1 to 4: strain AYE△*adeG*; Lanes 5 to 8: strain AYE; Lanes 13 to 16: strain AYE△*adeIJK*; Lanes 17 to 20: strain AYE. The confirmation of △*adeFGH* (ca. 5500 bp deletion) with primers adeG RT F/R yielded no PCR product in Lane 1 (strain AYE△*adeFGH*) and a product in Lane 5 (AYE); while the confirmation of △*adeG* with primers adeG up F (NotI)/adeG down R (SphI) produced a product in Lane 2 (strain AYE△*adeG*) that is ca. 5500 bp smaller than that in Lane 6 (strain AYE). The confirmation of △*adeIJK* (ca. 3300 bp deletion) with primers adeJ F/R (Lanes 13 and 17) or primers adeJ up F (PstI)/adeJ down R (SphI) (Lanes 14 and 18) yielded, respectively, products in Lanes 13 and 14 (strain AYE△*adeJ*) that are ca. 3300 bp smaller than those in Lanes 17 and 18 (strain AYE). Lanes 3, 7, 15 and 19 used primers AYE csy F/R to produce a PCR product that ensured bacterial cells tested as *A. baumannii* AYE and its derivatives. Lanes 9-12 and 20-24 used primers p130-F/R with no PCR products detected to ensure that the pMo130-Tel^R^ vector was eliminated from the chromosome after the second crossover on the selection plate.


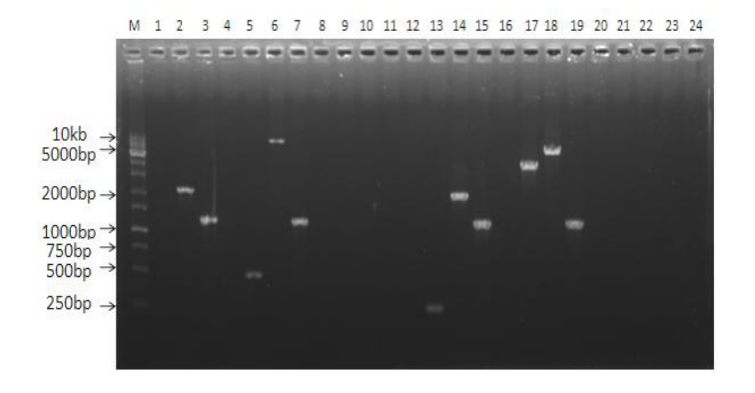


**Fig S4.** Twitching motility comparison of *A. baumannii* AYE and its six RND efflux system gene deletion mutants on soft tryptone agarose plates in the presence of 1% NaCl. The results are representative of three independent experiments showing similar results. NaCl at 0.25%, 0.5% and 1% had no significant effect on the motility (Table S3).


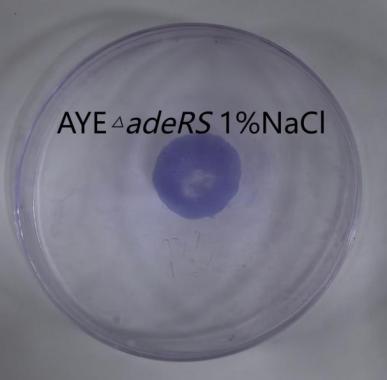

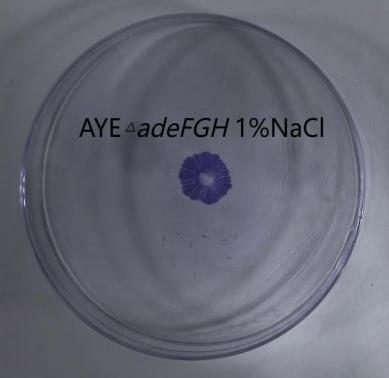

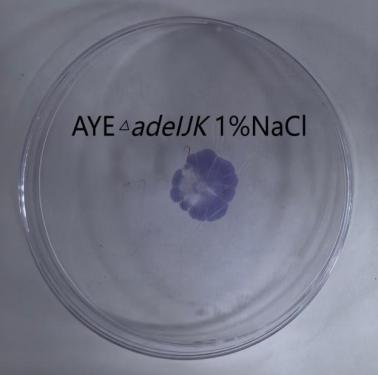

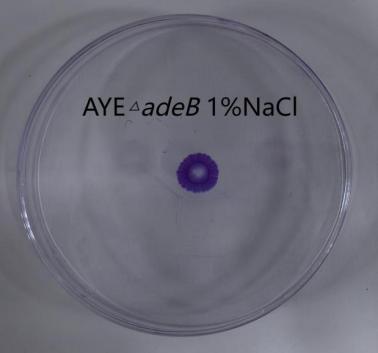

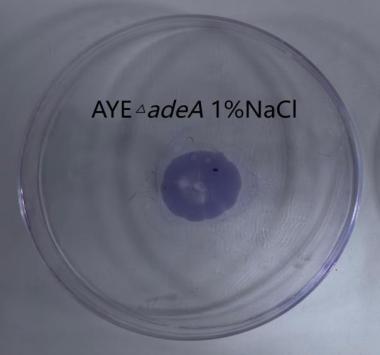

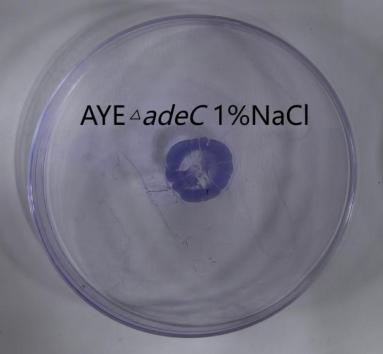

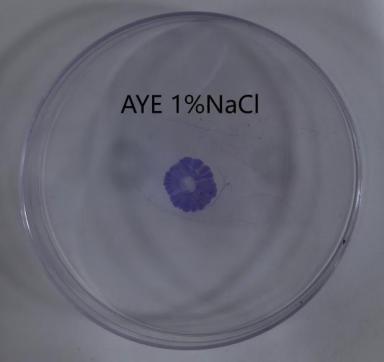


**Fig S5.** Swarming motility comparison of *A. baumannii* AYE and its six RND efflux system gene deletion mutants on soft tryptone agar plates and the effect of NaCl (0.5% [A], 0.25% [B] and 0.125% [C]). The results are representative of three independent experiments showing similar results.

**A. 0.5% NaCl**


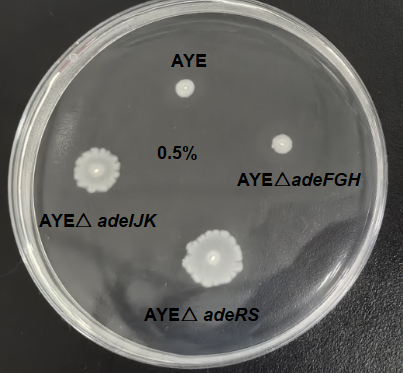

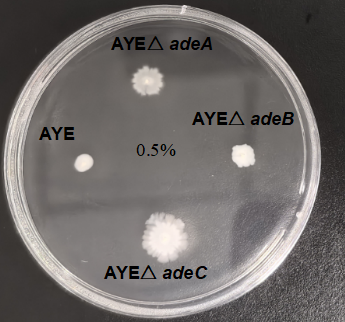


**B. 0.25% NaCl**


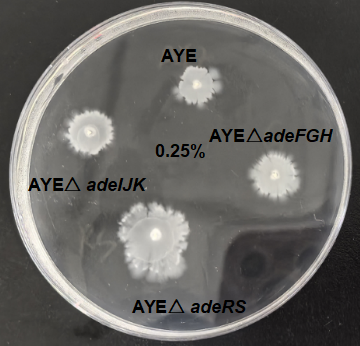

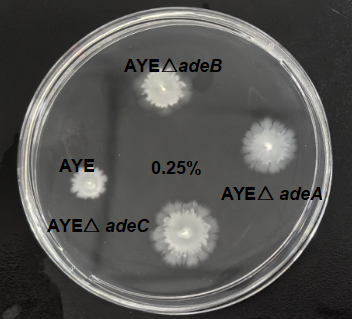


**C. 0.125% NaCl**


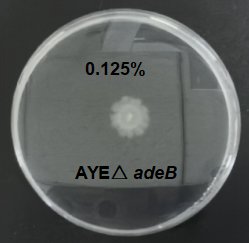

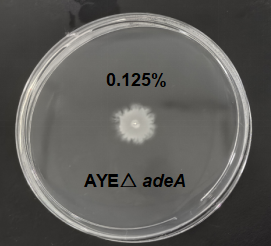

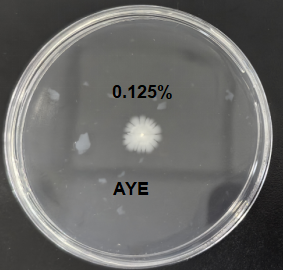


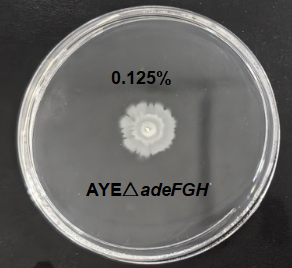

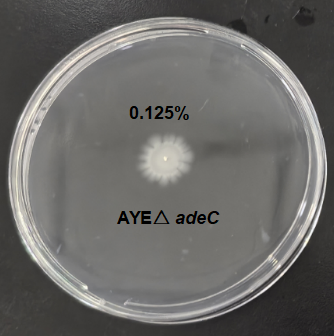

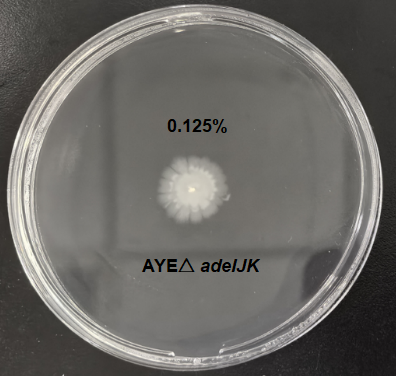

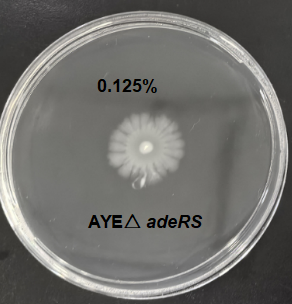


**Fig S6.** Susceptibility of *A. baumannii* AYE and its six RND efflux system gene deletion mutant strains to normal human serum. No significant (ns) differences were observed among bacterial cells treated with heat-inactivated serum and with serum.


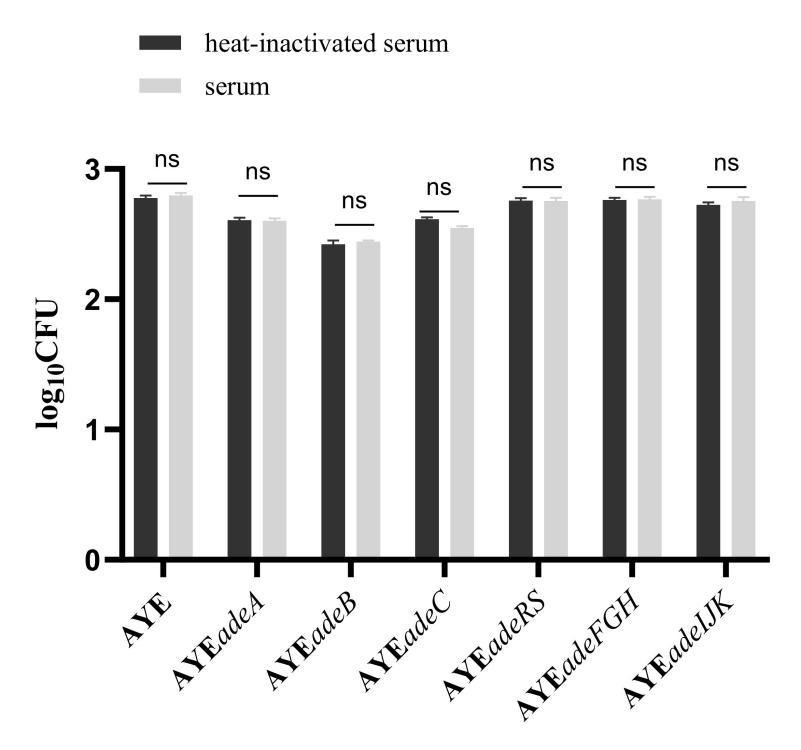


**Table S1.** Target genes and the primer sequences used for gene expression experiments.

| **Target gene** | **Annotation** | **Primer sequence (5’→3′)** | **qPCR product size (bp)** | **Reference** |
| --- | --- | --- | --- | --- |
| *adeA* | Membrane fusion protein AdeA of RND AdeABC pump system | Forward: AAATAGTGACAAAACCGTTCG  Reverse: TACCCGGAAGTAATTTTCGTT | 158 | (22) |
| *adeB* | Transporter AdeB of AdeABC | AAAGGTATTGGCTACGAGTGG  TGCCCAGCTTTCATAGAGTG | 132 | (22) |
| *adeC* | Outer membrane protein AdeC of AdeABC | TAAATCGGCAATCGTATCTCGT  ATGACCTGTACTAATTTCGGAT | 198 | (22) |
| *adeR* | Response regulator AdeR of AdeRS two-component system | CTTCCCAACCGTTTAATTCG  TTATTTAAAACGTGAAGGCAT | 131 | (28) |
| *adeS* | Sensor AdeS of AdeRS | ATCGCATTATCAATTAAAGCA  CTTAGTAGAGAACCAGCAAC | 198 | (28) |
| *adeG* | Transporter AdeG of RND AdeFGH pump system | GCGTTGCTGTGACAGATGTT  TTGTGCACGGACCTGATAAA | 104 | (39) |
| *adeJ* | Transporter AdeJ of RND AdeIJK pump system | GGTCATTAATATCTTTGGC  GGTACGAATACCGCTGTCA | 222 | (24) |
| *abaI* | Quorum sensing system auto-inducer synthase | GACTGCTAGAGGAAGGCGGATTTG  AGACTACTACCCACCACACAACCC | 148 | (44) |
| *abaR* | Quorum sensing system auto inducer synthase receptor | TAAATGTCGGTTGGGCTCAGTCAAG  GCTGGAATGCACTGTTTGAGTCAAC | 149 | (44) |
| *bfmR* | Response regulator BfmR of BfmRS two-component system | GTG AAGTTCGCCCACACTAT  GCACCCATTTCCAAACCAAG | 91 | (46) |
| *bap* | Biofilm-associated protein Bap | AATGCACCGGTACTTGATCC  TATTGCCTGCAGGGTCAGTT | 205 | (45) |
| *csuE* | Adhesin CsuE of the Csu pilus subunits CsuA/B/C/E | ACCTTTCTACATACGGCTTCC  CAACTGCGGGTACAGAATAGA | 104 | (47) |
| *ompA* | Outer membrane protein OmpA | AGCATAAAGAAGCTACACCTGC  AAAGTCGCCAAGAAACCTTGAT | 154 | (48, 49) |

| *pgaA* | Outer membrane protein PgaA | TTGATCCAGATGATTAGCGTAGG  AGTCAGGCTAAGGGTGTAGATA | 99 | (50) |
| --- | --- | --- | --- | --- |
| *16S rRNA* | Small subunit ribosomal RNA | ATGTGAAATCCCCGAGCTT  TACGCATTTCACCGCTACAC | 101 |  |

**Table S2.** Primers used in the construction of the RND efflux system gene deletion mutants of *A. baumannii*.

| **Target gene** | **Primer name (Restriction enzyme)** | **Primer sequence (5’→3′) (Restriction enzyme site underlined)** |
| --- | --- | --- |
| *adeA* | adeA up F (NotI) | ATATGCGGCCGCACATGATCACGGGAGTCTG |
|  | adeA up R | ACTGTCCAAACCTAGTGAC |
|  | adeA down F | TGTCACAATTTTTATTCGTCGTCC |
|  | adeA down R (BamHI) | CGCGGATCCCTTCAACCTGTAAGCCTTGC |
|  | adeA middle F | CTAGGTTTGGACAGTTGTCACAATTTTTTATTCG |
|  | adeA middle R | CGAATAAAAAATTGTGACAACTGTCCAAACCTAG |
| *adeB* | adeB up F (NotI) | ATATGCGGCCGCCCACCACCGGCTAAAGTCAGT |
|  | adeB up R | GGTTGCGCCCCCTCAGCTATA |
|  | adeB down F | GTTCAATGCATCAGGGGAATAC |
|  | adeB down R (BamHI) | AGCTGGATCCGCTTTAAGGTCTGGACGATG |
|  | adeB middle F | TAGCTGAGGGGCGCAACCGTTCAATGCATCAGGGGAATAC |
|  | adeB middle R | GTATTCCCCTGATGCATTGAACGGTTGCGCCCCCTCAGCTA |
| *adeC* | adeC up F (NotI) | ATATGCGGCCGCGGAGCAATCATTGCCATTATG |
|  | adeC up R | AGTATTCTCCAAATAAAGTAAATTTTGAGC |
|  | adeC down F | TAAAGTTTCACTCTTAAAAAAATAAGCTG |
|  | adeC down R (BamHI) | CGCGGATCCCAATTAACCGTTGCAGTCGC |
|  | adeC middle F | CTTTATTTGGAGAATACTTAAAGTTTCAGTC |
|  | adeC middle R | GAGTGAAACTTTAAGTATTCTCCAAATAAAG |
| *adeRS* | adeRS up F (NotI) | ATATGCGGCCGCGCCGAGCACAGTCCATTTAC |
|  | adeRS up R | TTCGCTAAATTAAAAAATCTTAGAGTTAAAGTGC |
|  | adeRS down F | AGAAAATCTGGCTATAGAAAGTGC |
|  | adeRS down R (BamHI) | CGCGGATCCCTCGAACTGTTGCATATTGC |
|  | adeRS middle F | GATTTTTTAATTTAGCGAAAGAAAATCTGGC |
|  | adeRS middle R | GCCAGATTTTCTTTCGCTAAATTAAAAAATC |
| *adeA* | adeA 5’ UTR up F (NotI) | ATATGCGGCCGCCGAATAACACTCATGCCTTCAGG |
|  | adeA 5’ UTR down R (BamHI) | CGCGGATCCCTGCGGTTGAATGCTTAATACACTGAC |
| *adeC* | adeC 5’ UTR up F (NotI) | ATATGCGGCCGCCGAATAACACTCATGCCTTCAGCAACC |
|  | adeC 5’ UTR down R (BamHI) | CGCGGATCCGGAATATGAGATGTGATTGCAGGCTG |
| *adeG* | adeG up F (NotI) | TATGCGGCCGCATTAAGTTTTGAGCGTATAAGCTTC |
|  | adeG up R (BamHI) | GCTGGATCCATGAGAGTATTCAACAAAGT |
|  | adeG down F (BamHI) | TATGGATCCAAATATTGCTCGTAACAGAG |
|  | adeG down R (SphI) | TATGCATGCAAGACGTAACTGGTAAAGAT |
|  | adeG RT F | ACTTGTTGCAGTATTCGTAC |
|  | adeG RT R | TTAAGTGCAGTGTCACTCAT |
|  | adeG F | GCGATAAAGTCATTGTTGATG |
|  | adeG R | GTTCTGTGTAATCTGATATTGCTG |
| *adeJ* | adeJ up F (PstI) | TATCTGCAGGAAGTAGGTGTTATTGTTGC |
|  | adeJ up R (BamHI) | TCTGGATCCTAAGGTTTTGCTGATACTTC |
|  | adeJ down F (BamHI) | TATGGATCCCGTGAAAACCGATATACCAC |
|  | adeJ down R (SphI) | TCTGCATGCCCACCAGACTTAAATAGATC |
| Plasmid pMol130-Tel^R^ | pMol130-Tel^R^ F | GCGGGGGAGATTACAACTAC |
|  | pMol130-Tel^R^ R | GCGCGTTTCGGTGATGA |
|  | AYE plasmid p2 F | CCCCGATTTTTATTCTTTGATTAT |
|  | AYE plasmid p2 R | GCCGGCCCTGTTGGTATGAGTG |
|  | AYE plasmid p4 F | GGGTTTCAGGCGTGTTTTTTTATAG |
|  | AYE plasmid p4 R | TGCTTCATCCAGGCTTTCCATAG |
|  | AYE csy F | CGCCGCTGCAATGGATGTCTTTAAG |
|  | AYE csy R | GCCGGTCTGCTTCCATCTTTATCTA |

**Table S3.** Twitching motility of *A. baumannii* AYE and its six RND efflux system gene deletion mutant strains as well as the effect of NaCl.

| **Bacterial strain** | **Diameter (mm) (**$\bar{\boldsymbol{x}}$ **± s; n=3)** | | |
| --- | --- | --- | --- |
|  | **1% NaCl** | **0.5% NaCl** | **0.25% NaCl** |
| AYE | 13.41±0.86 | 12.45±0.75 | 13.58±0.62 |
| AYE△*adeA* | 19.55±0.54 | 18.97±0.97 | 18.92±0.47 |
| AYE△*adeB* | 9.31±0.80 | 9.25±0.69 | 8.98±0.68 |
| AYE△*adeC* | 18.54±1.20 | 18.26±0.63 | 19.09±0.35 |
| AYE△*adeRS* | 23.97±1.66 | 23.59±1.05 | 23.06±0.63 |
| AYE△*adeFGH* | 12.89±0.68 | 12.56±0.59 | 12.83±0.82 |
| AYE△*adeIJK* | 18.68±0.98 | 18.64±0.69 | 19.36±0.57 |

**Table S4.** Biofilm formation ability of *A. baumannii* AYE and its six RND efflux system gene deletion mutant strains ($\bar{x}$ ±s, n=5).

| **Bacterial strain** | **OD_570_/OD_600_** **value** | **Biofilm inhibition rate (%)** |
| --- | --- | --- |
| AYE | 0.544±0.018 | 0 |
| AYE△*adeA* | 0.363±0.028 | 33 |
| AYE△*adeB* | 0.322±0.011 | 41 |
| AYE△*adeC* | 0.376±0.033 | 31 |
| AYE△*adeRS* | 0.408±0.014 | 25 |
| AYE△*adeFGH* | 0.460±0.032 | 16 |
| AYE△*adeIJK* | 0.361±0.010 | 34 |

**Table S5.** Expression levels of virulence factor-related genes in *A. baumannii* AYE and its six RND efflux system gene deletion mutant strains.

| **Bacterial strain** | **Relative expression (2^-△△CT^) (**$\bar{\boldsymbol{x}}$ **± s)** | | | | | | |
| --- | --- | --- | --- | --- | --- | --- | --- |
|  | ***abaI*** | ***abaR*** | ***bap*** | ***bfmR*** | ***csuE*** | ***ompA*** | ***pgaA*** |
| AYE | 1.00 | 1.00 | 1.00 | 1.00 | 1.00 | 1.00 | 1.00 |
| AYE△*adeA* | 0.72±0.03 | 5.86±0.54 | 0.28±0.12 | 0.36±0.07 | 0.21±0.01 | 0.51±0.02 | 0.28±0.07 |
| AYE△*adeB* | 0.24±0.13 | 3.93±0.40 | 0.21±0.02 | 0.15±0.06 | 0.03±0.01 | 0.06±0.03 | 0.19±0.07 |
| AYE△*adeC* | 0.53±0.18 | 6.92±0.28 | 0.31±0.08 | 0.38±0.01 | 0.16±0.06 | 0.28±0.09 | 0.30±0.02 |
| AYE△*adeRS* | 0.52±0.04 | 8.49±0.67 | 0.18±0.03 | 0.31±0.02 | 0.44±0.05 | 0.88±0.10 | 0.26±0.07 |
| AYE△*adeFGH* | 0.80±0.16 | 2.14±0.07 | 0.99±0.16 | 0.93±0.03 | 0.92±0.20 | 0.15±0.02 | 0.78±0.06 |
| AYE△*adeIJK* | 0.47±0.05 | 6.68±0.38 | 0.19±0.01 | 0.45±0.03 | 0.39±0.03 | 0.52±0.12 | 0.41±0.03 |

**Table S6.** Antimicrobial specific resistance determinants of *A. baumannii* AYE based on the complete genome sequence (GenBank accession: NC_010410, as of July 15, 2024; Available at: *<https://www.ncbi.nlm.nih.gov/nuccore/NC_010410.1>*) (10).

| **Antimicrobial class** | **Resistance mechanism** | **Gene locus** |
| --- | --- | --- |
| β-Lactams | Extended-spectrum class A β-lactamase VEB-1 | ABAYE_RS17995 |
|  | Extended-spectrum class C β-lactamase ADC-11 | ABAYE_RS05990 |
|  | Oxacillin-hydrolyzing class D β-lactamase OXA-10 | ABAYE_RS17975 |
| Aminoglycosides | Aminoglycoside *N*-acetyltransferase AAC(3)-Ia | ABAYE_RS17770 |
|  | Aminoglycoside *N*-acetyltransferase AAC(6')-Ian | ABAYE_RS18030 |
|  | Aminoglycoside nucleotidyltransferase ANT(2'')-Ia (AadB) | ABAYE_RS17990 |
|  | Aminoglycoside nucleotidyltransferases ANT(2'')-Ia (AadB) (two enzymes) | ABAYE_RS17800 ABAYE_RS17970 |
|  | Aminoglycoside nucleotidyltransferase ANT(3'')-IIa | ABAYE_RS18565 |
|  | Aminoglycoside *O*-phosphotransferase APH(3')-Ia | ABAYE_RS17800 |
|  | Aminoglycoside *O*-phosphotransferase APH(3'')-Ib | ABAYE_RS18115 |
|  | Aminoglycoside *O*-phosphotransferase APH(6)-I | ABAYE_RS18110 |
| Fluoroquinolones | DNA gyrase subunit A (GyrA) | ABAYE_RS04860 |
|  | DNA topoisomerase (ATP-hydrolyzing) subunit B (GyrB) | ABAYE_RS00570 |
|  | DNA topoisomerase IV subunit A (ParC) | ABAYE_RS18255 |
|  | DNA topoisomerase IV subunit B (ParE) | ABAYE_RS01165 |
| Phenicols | Chloramphenicol efflux transporter CxpE | ABAYE_RS04965 |
|  | Chloramphenicol efflux major facilitator superfamily (MFS) transporter CmlA5 | ABAYE_RS17980 |
| Rifamycins | NAD^(+)^--rifampin ADP-ribosyltransferase Arr-2 | ABAYE_RS17985 |
| Tetracyclines | Tetracycline efflux MFS transporter Tet(A) | ABAYE_RS17875 |
|  | Tetracycline efflux MFS transporter Tet(G) | ABAYE_RS18065 |
|  | Tetracycline resistance MFS efflux pump | ABAYE_RS02365 |
